# Identification of Genic SSRs Provide a Perspective for Studying Environmental Adaptation in the Endemic Shrub *Tetraena mongolica*

**DOI:** 10.3390/genes11030322

**Published:** 2020-03-18

**Authors:** Zhenhua Dang, Lei Huang, Yuanyuan Jia, Peter J. Lockhart, Yang Fong, Yunyun Tian

**Affiliations:** 1 Inner Mongolia Key Laboratory of Grassland Ecology & Ministry of Education Key Laboratory of Ecology and Resource Use of the Mongolian Plateau, School of Ecology and Environment, Inner Mongolia University, Hohhot 010021, China; zhdang_1982@aliyun.com (Z.D.); huanglei_1996@163.com (L.H.); j773839714@163.com (Y.J.); 2 School of Fundamental Sciences, College of Sciences, Massey University, Palmerston North 4442, New Zealand; p.j.lockhart@massey.ac.nz (P.J.L.); r.fong@massey.ac.nz (Y.F.); 3 Ministry of Education Key Laboratory of Herbage & Endemic Crop Biotechnology, School of Life Sciences, Inner Mongolia University, Hohhot 010021, China

**Keywords:** genic SSR, polymorphic, environmental adaptation, transcriptome, *Tetraena mongolica*

## Abstract

*Tetraena mongolica* is a xerophytic shrub endemic to desert regions in Inner Mongolia. This species has evolved distinct survival strategies that allow it to adapt to hyper-drought and heterogeneous habitats. Simple sequence repeats (SSRs) may provide a molecular basis in plants for fast adaptation to environmental change. Thus, identifying SSRs and their possible effects on gene behavior has the potential to provide valuable information for studies of adaptation. In this study, we sequenced six individual transcriptomes of *T. mongolica* from heterogeneous habitats, focused on SSRs located in genes, and identified 811 polymorphic SSRs. Of the identified SSRs, 172, 470, and 76 were located in 5′ UTRs, CDSs, and 3′ UTRs in 591 transcripts; and AG/CT, AAC/GTT, and AT/AT were the most abundant repeats in each gene region. Functional annotation showed that many of the identified polymorphic SSRs were in genes that were enriched in several GO terms and KEGG pathways, suggesting the functional significance of these genes in the environmental adaptation process. The identification of polymorphic genic SSRs in our study lays a foundation for future studies investigating the contribution of SSRs to regulation of genes in natural populations of *T. mongolica* and their importance for adaptive evolution of this species.

## 1. Introduction

*Tetraena mongolica* (2*n* = 2*x* = 28) [1], a monotypic species of the Zygophyllaceae family, is a xerophytic shrub that originated from the Tethys Ocean, and is now endemic to the Eastern Alxa–Western Ordos desert region in Inner Mongolia, China [2,3]. The area is characterized by hyper-drought conditions, low annual temperatures, and high soil salinity [4]. To adapt to the semi-desert environment, *T. mongolica* has evolved distinct survival strategies, such as an extensive root system, thick cuticles on its stems, and succulent leaves. *T. mongolica* has important ecological roles as a windbreak and in the fixation of local ecosystem soils [3]. Moreover, high levels of triacylglycerol in its stems makes it combustible even in the fresh state, so it has been used as firewood and is named locally as “oil firewood” [5,6]. *T. mongolica* is a typical species that grows in this area and is a good candidate for studies of plant origin, evolution, and adversity adaptation in such desert regions. Around these topics, a series of fundamental research investigations have been implemented, the results of which are of importance for comprehensively understanding the properties of *T. mongolica*. This pioneering work includes the studies of systematic, geographic, and taxonomic aspects [7,8,9]; morphology and anatomy [10,11,12]; physiological ecology [13,14,15,16]; chemometrics and biochemistry [17,18]; population genetics [5]; conservation biology [19,20,21]; abiotic stress response [3]; and medicinal phytochemistry [22,23]. In recent decades, the plant has suffered from serious habitat destruction in size and has even disappeared, leading to the general decline of the species and making the survival of *T. mongolica* much harder than before [24]. Correspondingly, one consideration for the ongoing survival of the species that has been paid much attention recently is an apparent decrease in genetic diversity due to loss of demographics, inbreeding depression, stochastic events, and other factors [5,24]. However, contrary to the assumption of diversity loss, it has been observed that the species has an intermediate or high level of genetic diversity as detected from analyses of inter-simple sequence repeats and nuclear microsatellites, respectively [5,24]. Therefore, further exploration of these hereditary characteristics and a potential link to adaptation strategies of the species is needed.

Genic simple sequence repeats (SSRs) (also called genic microsatellites or expressed sequence tag (EST)-SSRs), a relatively newly established concept, are simple sequence repeats located in genes, including protein-coding genes (CDSs) and their untranslated regions (UTRs) [25]. With the rapid accumulation of genetic data from expressed sequence tags (ESTs), a vast number of genic microsatellites have been identified and studied in many plants, especially for non-model species, which have a limited genomic background. Several outstanding characteristics of genic SSRs have led to these markers being widely used for the study of germplasm characterization [26], genetic mapping and breeding studies [27], and functional genome analysis [28]. The merits of these molecular resource include their high quality and relative ease-of-use for comparisons between genome counterparts, the high frequency and non-random distribution within transcribed regions of the genomes, and the transferability of markers to related species or genera [25]. It has been revealed that variations of these SSRs may change gene activity or regulate gene expression, thus affecting biological and cellular processes, such as protein structure, sensing and signaling, and gene transcription [29]. For example, CGG/CCG repeats have been associated with DNA methylation, indicating that this repeat may be the trigger for gene silencing [30]. ATTCT/AGAAT repeats can readily form unpaired regions in supercoiled plasmids, which have roles in the initiation of replication [31]. The GAA/TTC repeats form triplexes and related structures known as “sticky DNA”, which trap RNA polymerase on the template, thus blocking transcription elongation [32,33]. Therefore, genic SSRs have been proposed as evolutionary tuning knobs that may provide a molecular basis for fast adaptation to environmental changes in organisms [34]. Identification and in-depth study of these genomic elements, especially those in natural populations that inhabit ecologically heterogeneous and stressful environments, can provide new insights into the adaptive evolutionary mechanisms of plants [35,36].

Next-generation sequencing technologies have revolutionized studies in molecular ecology [37]. These technologies and relevant bioinformatics tools have helped in the identification of many genetic variations and provided insights into adaptive evolution, population genomics, and conservation genomics [37,38]. In this study, we sequenced, assembled, and compared the transcriptomes of six individual *T. mongolica* plants collected from heterogeneous habitats, and identified, validated, and analyzed the characteristics of polymorphic genic SSRs in this species. Our results will facilitate further studies into the functions of the identified SSRs, adaptive evolution mechanisms of *T. mongolica*, and the protection and exploration of the germplasm resources of this species.

## 2. Materials and Methods 

### 2.1. Plant Materials

Leaves of *T. mongolica* were collected from six natural populations in June 2015 from the Eastern Alxa–Western Ordos area in Inner Mongolia, China. Twenty individuals from each population were selected at intervals of 30 m to avoid sampling progeny from the same maternal parent. Detailed geographical information about the sampling location is given in Table 1. After collection, the leaf samples were snap frozen in liquid nitrogen, then kept in a −80 °C refrigerator until they were transported to the laboratory.

### 2.2. RNA Preparation, cDNA Library Construction, and Sequencing

*T. mongolica* leaves (one sample per population) were used for RNA isolation. Total RNA was extracted using a Plant Plus RNA Kit (DP437, Tiangen, Beijing, China), according to the manufacturer’s instructions. The extracted RNA was treated with deoxyribonuclease I (TaKaRa Bio Inc., Otsu, Shiga, Japan) for 30 min at 37 °C to remove residual DNA. RNA quality was monitored using an Agilent 2100 Bioanalyzer with a minimum RNA integrity number value of 8.0. Six cDNA libraries (D1–D6) were constructed and sequenced on a paired-end flow cell device using an Illumina HiSeq^TM^ 4000 sequencer at Biomarker Biotechnology Co., Ltd (Beijing, China). The sequencing data were called and quality controlled through the Illumina data processing pipeline.

### 2.3. De novo Assembly and Sequence Clustering

The adaptor reads, low-quality reads, and reads in which the proportion of “N” was greater than 5% were filtered to obtain the clean reads. *De novo* transcriptome assembly was performed for each sample using Trinity software (v2.4.0). TGICL v2.1 (http://sourceforge.net/projects/tgicl/files/tgicl%20v2.1/) was used to cluster the homologous transcripts and form an unextendible unigene. Finally, to eliminate redundancy and splicing among the six assembled transcriptomes, we further merged the unigenes to obtain non-redundant unigenes (all-unigenes).

### 2.4. Identification of Potential Polymorphic Genic SSRs

CandiSSR was used to identify potential polymorphic genic SSRs among the six unigenes datasets of *T. mongolica* [39]. The parameters used in the pipeline were: flanking sequence length 100 bp, blast e-value cutoff 1.0 × e^−10^, blast identity cutoff 95%, and blast coverage cutoff 95% [39,40]. The SSR search criteria were designed for di-, tri-, tetra-, penta-, and hexa-nucleotide repeats, with a minimum repeat number of six, five, five, four, and four, respectively, while SSRs with mono-nucleotide repeats and motif bigger than six nucleotides were not examined in this study. Primers for the identified SSRs were designed automatically in the pipeline using Primer3 [41].

### 2.5. Location Prediction and Functional Annotation of the Identified SSRs

We first determined the protein-coding sequences (CDSs) of the SSR-containing sequences. The sequences were aligned to protein databases in the priority order of NR, Swiss-Prot, KEGG, and COG using BLASTX (http://blast.ncbi.nlm.nih.gov/Blast.cgi), with a significance threshold e-value <1.0 × 10^−5^. The highest ranked proteins in blast results were used to predict the CDSs of the SSR-containing sequences. The results were then used to identify the distributions of the SSRs. For unigenes with full-length CDSs, the locations of the SSRs were analyzed based on the relative positions of the SSRs with respect to the start (ATG) and stop (TAA, TAG, TGA) codons. For unigenes with partial CDSs, we manually performed nucleotide blast analysis to identify full-length homologous genes and then predicted the locations of the SSRs according to their positions in the query sequences. To obtain functional information about the genic-SSR-containing unigenes, gene ontology (GO) functional categories were identified using the Blast2GO software v2.5.0 (http://www.blast2go.com/b2ghome) and pathways were assigned by sequence searches against the KEGG database using the BLASTX algorithm, with an e-value threshold of 1.0 × 10^−5^.

### 2.6. SSR Polymorphic Validation

Genomic DNA was extracted from *T. mongolica* leaves (eight individuals per population) using a Plant Genomic DNA kit (DP305, TianGen, Beijing, China). DNA quality was checked on 1% TAE agarose gels and NanoDrop 2000c (Gene Company Limit, Beijing, China). PCR amplifications were performed on the ABI2720 Thermal cycler in 25 μl reaction mixtures that included 1 μl of template DNA (50 ng/μL), 12.5 μL of Premix Taq (TaKaRa Biotechnology Co., Dalian, Liaoning Province, China), 0.5 μl (10 pM) of forward primer, 0.5 μl (10 pM) of reverse primer, and ddH_2_O. PCR amplifications were carried out as follows: 5 min at 94 °C, followed by 30 cycles of 30 s at 94 °C, 30 s at the primer-specific annealing temperature (Table 2), 30 s at 72 °C, and the final extension step at 72 °C for 10 min. For the successfully amplified primer pairs, the 5′ end of each forward primer was tagged with one of three fluorescent dyes (6-carboxy-fluorescine, hexachloro-6-carboxy-fluorescine, and 6-carboxy-X-rhodamine) and used for amplifications with the same protocol. The labeled PCR products were analyzed on an ABI 3730xl DNA analyzer with a GeneScan 500 LIZ Size Standard (Applied Biosystems, Beijing, China). Allele sizes were called using GeneMarker v2.6.0 (SoftGenetics, State College, Pennsylvania, USA). Several genetic parameters, including mean number of alleles per locus (*N_A_*) and observed and expected heterozygosity (*H_O_* and *H_E_*), were estimated across all the identified loci using GenAlEx software (v6.502) [42]. Polymorphism information content (*PIC*) of each locus was measured by PowerMarker program (v3.0) [43].

### 2.7. Data Availability

The raw sequencing data have been deposited in the Sequence Read Archive under accession number PRJNA597711.

## 3. Results

### 3.1. Sequencing Outputs and de novo Assembly

A total of 51.48 gigabases of clean data were obtained from the six cDNA libraries. As shown in Table 2, the clean reads in all samples had an average CG content of 44.24%, and the quality of these data were quite high, with an average Q_20_ percentage nearly of 97.15%. After *de novo* assembly, 73,977 to 92,301 unigenes in the six datasets were generated, and the mean length and N_50_ were 806 bp and 1536 bp, respectively. Finally, the assembled unigenes of the six transcriptomes were further clustered into a total of 119,603 all-unigene sequences, which had an average length of 1098 bp and average N_50_ value of 1843 bp.

### 3.2. Identification of Polymorphic SSRs 

The CandiSSR analysis detected 811 polymorphic SSRs that were present in almost every dataset. The polymorphic levels of each SSR in these datasets were random (Table S1). The identified SSRs represented 89 motif types in 646 of the all-unigene sequences. The most common SSRs were tri-nucleotide repeats (522, 64.36%), followed by di-nucleotide repeats (261, 32.18%); quad-, penta-, and hexa-nucleotide repeats were far less frequent. Among the tri-nucleotide repeats, AAC/GTT (81, 15.52%) was the most abundant, followed by AGC/GCT (52, 9.96%), ACT/AGT (48, 9.20%), and AAG/CTT (43, 8.24%). Among the di-nucleotide repeats, AT/AT (119, 45.60%) was the most abundant, followed by AG/CT (99, 37.93%) and AC/GT (43, 16.48%).

### 3.3. Location Prediction and Frequency Analysis of the Polymorphic SSRs

**Figure 1:**
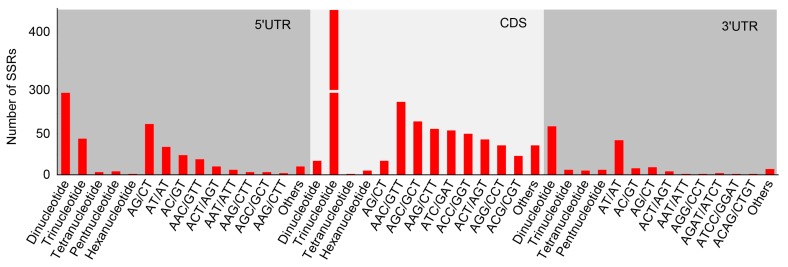
Distribution analysis of the identified genic simple sequence repeats (SSRs). The *y*-axis indicates the numbers of identified genic SSRs; the *x*-axis indicates the distribution of the polymorphic genic SSRs and motif sequence types.

Among the polymorphic SSR-containing sequences, 620 were aligned with known proteins and 763 SSRs were embedded in these transcripts. Of these SSRs, 718 were located in the coding and untranslated regions of 591 unigenes. Among them, 172, 470, and 76 SSRs in 150, 372, and 69 unigenes were distributed in 5′ UTRs, coding regions, and 3′ UTRs, respectively (Table S2). Most of the tri-nucleotide repeats (90.12%) were located in CDSs, while most of the di-nucleotide repeats (91.33%) were located in UTRs. In the CDSs, AAC/GTT, AGC/CTG, AAG/CTT, ATC/ATG, AAG/CTT, ACC/GGT, and ATC/GAT were relatively abundant, together accounting for 62.55% of all the SSRs located in CDSs. AG/CT (62, 36.05%) was the most abundant repeat in 5′ UTRs and AT/AT (42, 55.26%) was the most abundant repeat in 3′ UTRs (Figure 1).

### 3.4. Functional Annotation of the Polymorphic SSR-Containing Sequences

**Figure 2:**
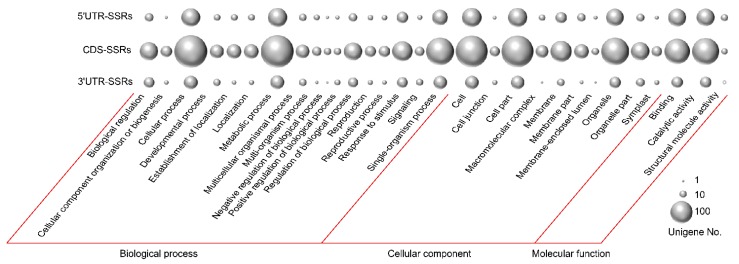
Gene ontology (GO) functional annotation of the polymorphic SSR-containing sequences. The three lines of bubbles represent SSRs that were located in the 5′ untranslated regions (UTRs), protein-coding sequences, and 3′ UTRs, respectively. GO terms that contained unigenes more than or equal to ten in one of the gene regions are shown in the figure. The relative number of unigenes assigned to each term is indicated by the size of each bubble. The grey hollow circle indicates no unigene was assigned to the relevant GO term.

Based on the sequence similarity, the 424 unigenes containing 525 SSRs were mapped to 3561 terms in the GO database by blast. The terms under the three main GO categories were divided among 45 sub-categories. For 5′ UTRs that contained SSRs, the most abundant terms in each main categories were “metabolic process”, “cell”, and “binding”, respectively. For CDSs that contained SSRs, “cellular process” was the most abundant term, followed by “metabolic process”, “cell”, “cell part”, and “single-organism process”. For the 3′ UTRs that contained SSRs, the top three terms were “metabolic process”, “cellular process”, and “single-organism process” (Figure 2, Table S3).

**Figure 3:**
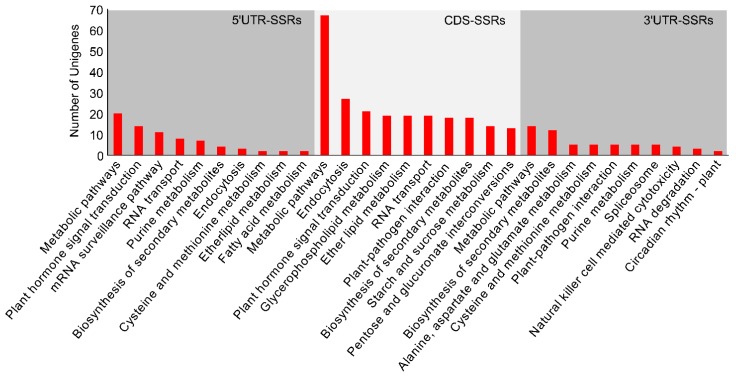
KEGG functional classification of the SSR-containing sequences. The *y*-axis indicates the numbers of SSR-containing sequences enriched in KEGG pathways; the *x*-axis indicates the top ten enriched pathways assigned to the 5′ UTR, CDSs, and 3′ UTR SSR-containing sequences.

Pathway analysis assigned 228 genic-SSR-containing sequences to 77 KEGG pathways (Figure 3, Table S4). For the 5′ UTRs, CDSs, and 3′ UTRs that contained SSRs, “metabolic pathways” was the most common and abundant term, followed by “plant hormone signal transduction”, “endocytosis”, and “biosynthesis of secondary metabolites” in each gene region.

### 3.5. Validation of the Polymorphic SSRs

**Figure 4:**
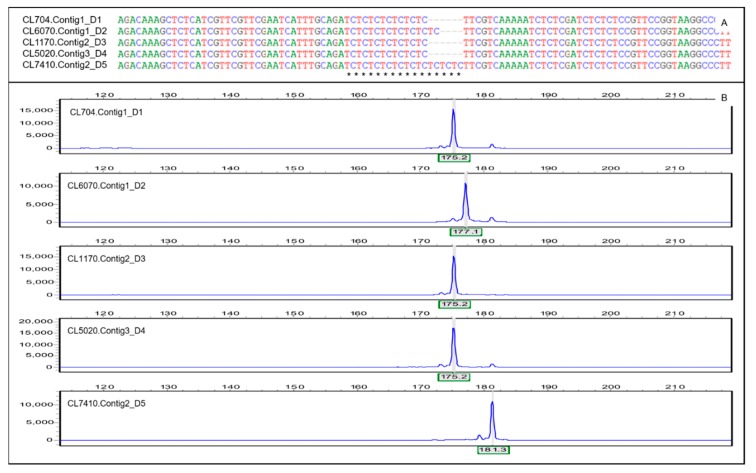
Representative polymorphic SSRs identified by CandiSSR and capillary electrophoresis. (**A**) Five unigenes assembled in the D1–D5 cDNA libraries that correspond to a polymorphic SSR identified by CandiSSR [39]. Multiple sequence alignment was performed using the Bioedit software (v7.0.9) [44]. (**B**) The capillary electrophoresis results for the polymorphic SSRs in (**A**).

Primer pairs were successfully designed for 795 identified SSRs using their flanking sequences (Table S5). To validate the effectiveness of these primers, 20 of the identified polymorphic SSRs were selected randomly and validated by PCR. Seventeen SSRs were successfully amplified with polymorphic loci. The loci size polymorphism levels were further assessed by capillary electrophoresis (Figure 4). A total of 76 alleles were detected with 2 to 7 (average 4.471) alleles obtained per locus (Table 3). The expected heterozygosity (*H_E_*), ranged from 0.274 to 0.774 and the observed heterozygosity (*H_O_*) from 0.167 to 0.729, with mean values of 0.587 and 0.418, respectively. The polymorphism information content (*PIC*) value ranged from 0.240 to 0.728, with an average value of 0.531 (Table 3).

## 4. Discussion

### 4.1. Identifying and General Profiling of Genic SSRs in T. mongolica Transcriptomes

SSRs have been recognized as an efficient tool for linking phenotypic and genotypic variations. They have been used widely in plants, for linkage map development, quantitative trait loci mapping, marker-assisted selection, parentage analysis, cultivar fingerprinting, genetic diversity studies, gene flow, and evolutionary studies [25,37]. Although microsatellite markers are versatile, developing polymorphic and robust SSR markers has been the main limitation for their use, especially for species that lack genomic background information. In recent years, next-generation sequencing technologies and a variety of computer programs (e.g., MISA [45], SSRFinder [46], SSRIT [47]) have promoted the identification of SSRs in model and non-model organisms [37]. However, the downstream development and identification of usable and researchable polymorphic SSRs is still a manual and time-consuming process [39]. Recently, an integrated procedure called CandiSSR was developed and used to obtain polymorphic SSRs in silico using genomic or transcriptomic sequence data [40]. In the present study, using the bioinformatics pipeline, we identified 811 potential polymorphic SSRs in six sequenced transcriptomes of *T. mongolica*. By comparing our finding with some other SSR development projects performed using CandiSSR, we found a similar polymorphic genic SSRs generation rate among these different studies. For example, 497 polymorphic genic SSRs were identified in the comparison of the transcriptomes representing the northern and southern population of *Parrotia subaequalis* [40]; and 1663 polymorphic EST-SSRs were identified by comparing the transcriptomes of tea plant and 19 *Camellia* species [48]. The identified SSRs in these studies were relatively small. One reason was probably attributed to the search criterial used in CandiSSR (SSRs with mono-nucleotide repeats and motif bigger than six nucleotides were excluded). Another reason was that the pipeline was designed to identify polymorphic SSRs directly based on the transcriptome or genome sequences comparison, thus the SSRs number would be significantly lower when compared to those SSR mining projects that were performed using only one dataset. Experimental validation showed that 17 of 20 randomly selected SSRs had 2 to 7 (average 4.471) polymorphic alleles per locus. The average *H_E_* and *H_O_* were 0.587 and 0.418, respectively (Table 3 and Figure 4). Although these genic SSRs may have low levels of polymorphism compared with the levels in genome SSRs (mean number of alleles per locus was 26.0, and mean *H_E_* and *H_O_* were 0.868 and 0.840, respectively [24]) in the *T. mongolica* genome, the genic SSRs have some intrinsic advantages (e.g., high transferability and functional importance) that make them a valuable resource that can be used for genetic and evolutionary studies of the species or its phylogenetically closed species and genus, such as species *Larrea tridentata* and genus *Viviania marifolia* [8,9]. In addition, Zhi et al. (2018) indicated that the highly polymorphic nuclear microsatellites might be over-estimating the genetic diversity of the present demography of *T. mongolica* [24], thus we considered that a rational genetic variation reserve could further be evaluated in *T. mongolica* by combing genetic data obtained from either the nuclear or genic SSRs. However, analyses of gene regions rather than non-coding genome regions evenly revealed a high level of genetic diversity in this endangered species [5,24], suggesting that the genome of the species is highly heterozygous to some extent and saturated with plenty of genetic variations that are potentially important for environmental adaptation and ongoing survival of the species. For the other three unamplified SSRs, we have speculated several possible reasons that might explain failure of the validations. Firstly, the PCR conditions might not have been suitable enough for amplifying the three SSRs. Secondly, the specificity of the primers was low. Thirdly, the primer binding sites may cross the boundary between introns and exons of the SSR-containing genes. Overall, whilst some markers could not be validated for technical reasons, we nevertheless were able to conclude that a large number of the identified SSRs in the present study are probably polymorphic and suitable for further study in *T. mongolica* transcripts. 

Strong evidence has shown that SSRs are non-randomly distributed across CDSs and UTRs [29]. In many species, exons contain many more tri- and hexa-nucleotide SSRs than di- and tetra-nucleotide SSRs [49,50]. This distribution bias may be explained by the low number of non-trimeric SSRs found in coding regions, possibly caused by frameshift mutations [51], that might be related to features of adaptation or responses to environment stress, as was previously found in *Escherichia coli* K12 [35]. In *T. mongolica*, the distribution analysis of transcribed sequences showed that > 90.12% of the identified tri-nucleotide SSRs were located in coding regions, whereas 91.33% of the di-nucleotide SSRs were distributed in UTRs (Figure 1). The SSRs in transcribed regions also showed bias to a specific nucleotide composition [29]. It was reported that the UTRs, in particular the 5′ UTRs, were strongly biased toward AG/CT in *Arabidopsis thaliana* [49,52]. In this study, almost all the identified AG/CT repeats were distributed in 5′ UTRs and most of the AT/AT repeats were located in 3′ UTRs. The SSRs in coding regions also exhibited a strong bias toward specific triplet repeats, such as AAG, AAC, ATC, AGC, AGG, and ACG, and these triplet repeats are known to be the most common repeats in the exons of dicotyledonous plants [53]. As shown in Figure 1, our results are consistent with these observations, so the identified SSRs in *T. mongolica* may have similar functions to their counterparts.

### 4.2. Probable Functions of Genic SSRs in the Environmental Adaptation of T. mongolica

Habitat heterogeneity and environmental pressures cause various stress responses that involve changes in gene transcription and translation in multiple cellular processes and metabolic pathways, and plants have evolved effective and efficient strategies to adapt to environmental changes for their survival [54,55]. Although narrowly distributed in the Ordos desert region, the eco-physiology characteristics of *T. mongolica* populations in their heterogeneous habitats (e.g., upland, hill, tableland, high plain) are different. Previous studies have shown that *T. mongolica* populations have different water parameters (water potential in saturated point, water potential in turgor loss point, available water capacity), endogenous hormones (indole-3-acetic acid, abscisic acid, gibberellin, zeatin riboside), antioxidant activities (superoxide dismutase, peroxidases, catalases, glutathione reductase), and photosynthesis, which suggests that the populations have evolved distinct adaptative mechanisms under heterogeneous growth conditions [14,15,16]. In recent decades, anthropogenic disturbances such as mineral exploitation, road building, overgrazing, and farming have led to habitat isolation, thus further accelerating the differentiation and survival adaptation of *T. mongolica* [19,20]. Genic SSRs have been proposed as fast-evolving genomic elements that might be of importance for adaptive evolution [34,36]. Therefore, it is reasonable to assume that the genes that contain polymorphic SSRs are important drivers of a plant’s response to environmental pressure and fluctuations. In the present study, we identified 646 transcripts that contained 811 polymorphic SSRs that may play significant roles in the local adaptation processes of *T. mongolica*. Of the SSR containing sequences, the majority (620) returned at least one blast hit with the known protein database at the e-value > 1.0 × 10^-5^, while the remaining 26 unigenes did not match known genes, suggesting that these unannotated unigenes might represent unique genetic resources of *T. mongolica*. Substantial data indicate that SSR expansion or contraction may affect gene expression and activity, resulting in concomitant physiological consequences and observable phenotypic changes [29,55]. Variations in 5′ UTRs have been found to regulate gene expression by affecting transcription and translation. In coding regions, variations can lead to a gain or loss of gene function via frameshift mutations or expanded toxic mRNA, and in 3′ UTRs variations can cause transcription slippage and produce expanded mRNA, which can disrupt splicing and disrupt other cellular functions [25,29]. Variations in CT repeats in the 5′ UTR of the starch-synthesis genes in waxy rice (*Oryza sativa*) were correlated with the physicochemical properties of starch [56]. Changes in the number of TGA repeats in the cellulase synthase gene (*PtoCesA10*) in *Populus tomentosa* resulted in an insertion mutation in the coding region and the addition of aspartate to the polypeptide chain of the enzyme, which is uniquely associated with the lignin content [57]. Myotonic dystrophy type 1 is caused by CTG repeat expansions in the 3′ UTR of *DMPK* [58,59]. In our analysis, 150, 372, and 69 unigenes were found to contain 172, 470, and 76 SSRs in 5′ UTRs, coding regions, and 3′ UTRs, respectively (Table S2). The GO functional annotations showed that the identified SSR-containing sequences were enrichened in “cellular process”, “metabolic process”, “cell”, “cell part”, and “single-organism process” terms under the three main GO categories, suggesting such groups of *T. mongolica* genes might first be regulated by SSRs mutations under the heterogeneous environments (Figure 2, Table S3). The KEGG analysis showed that among the SSR-containing 5′ UTRs and CDSs, 35 were distributed in “plant hormone signal transduction” (Figure 3, Table S4), implying that the regulation of endogenous hormones may play significant roles in the local adaptation of *T. mongolica*. This prediction is partially supported by the eco-physiology observations described above [14,15,16]. In future studies, we plan to further investigate how changes in the SSR repeats in natural *T. mongolica* populations impact gene functions and clarify the probable functions of these SSRs, which would provide hints for the study of environmental adaptation of the species and information to answer the question of whether genic SSRs play significant roles in adaptive evolution.

## Figures and Tables

**Table 1:** Location information for six population of *T. mongolica.*

Population	Longitude (E)	Latitude (N)	Altitude (m)	Habitats	Soil Water Content (%) *
D1	106°53′43″	39°21′57″	1212.5	Foothills	5.48
D2	106°53′52″	39°22′30″	1185.6	Tableland	5.24
D3	106°53′31″	39°29′52″	1216.5	Foothills	4.93
D4	107°05′45″	40°14′58″	1150.5	Tableland	3.89
D5	106°52′07″	40°08′03″	1036.9	Sandy Land	2.43
D6	106°55′07″	40°08′02″	1049.5	Piedmont Plain	2.43

*Cited from [16].

**Table 2:** Summary of sequencing and assembly results.

	CR (No.)	CN (nt)	Q_20_ (%)	GC (%)	Ug (No.)	ML (bp)	N_50_ (bp)
D1	53,284,254	7,992,638,100	97.22%	43.76%	80,409	791	1499
D2	55,473,386	8,321,007,900	97.19%	43.99%	80,829	824	1579
D3	64,363,372	9,654,505,800	96.88%	45.15%	77,641	786	1516
D4	52,017,954	7,802,693,100	96.94%	44.27%	84,673	851	1600
D5	63,352,430	9,502,864,500	97.36%	43.72%	92,301	794	1534
D6	54,704,688	8,205,703,200	97.31%	44.54%	73,977	788	1489
All					119,603	1098	1843

CR, CN, GC, and Ug represent clean read, clean nucleotide, GC content, and unigenes, respectively. Q_20_ represents the clean reads that had Phred-like quality scores at the Q_20_ level (an error probability of 1%). ML represents the mean length of assembled sequences and N_50_ indicates that 50% of the assembled bases were incorporated into sequences with a length of N_50_ or longer.

**Table 3:** Characteristics of 17 validated microsatellites for *T. mongolica.*

Gene ID	PS (5′–3′)	RM	AS	T_a_ (°C)	PF	*N_A_*	*H_O_*	*H_E_*	*PIC*
CL3279.Ct4	F: GTAGTACTACTGCTGCATCGTATCCT R: CAACCCTATCTTCATCATCATCG	TGC	101–113	54	protoporphyrinogen oxidase, *Vitis vinifera*	4	0.436	0.510	0.458
CL4993.Ct1	F: ACTCCTCTCATCCATCCATTAAG R: GGAGTTTAACGCTGTCATTGTG	TC	102–114	55	-	6	0.500	0.695	0.644
CL6111.Ct3	F: TGGAGTCTGAAGGCAGTGAG R: ACTTGAACTTCTTGATTCCACC	TAG	118–124	55	-	3	0.167	0.590	0.508
CL8609.Ct3	F: GCATTAGAGGAGCGAATCGAAG R: GCCTCGCTTCTCATTTCTCAAC	GA	164–174	59	-	6	0.417	0.617	0.584
CL8025.Ct4	F: CATCGCCGCCTTTCATAGAC R: GACGCTTAGAATTGGAAGATGATG	TC	175–181	55	cyclin-dependent kinase G-2-like, *Citrus sinensis*	3	0.354	0.472	0.422
Ug20261	F: GGGGAAAGATGCTGTTATGGAG R: TAGCATCCGAGCCACTACCAC	AGG	186–195	59	-	4	0.500	0.625	0.552
CL6305.Ct2	F: CGCTTGCTTTAACGACGAACC R: TGTGGTGGGTCGGATGATGTT	GCA	176–188	55	serine/threonine-protein kinase RIO1-like isoform X1, *Citrus sinensis*	5	0.279	0.686	0.621
CL7264.Ct1	F: GTTGTGGCGGCGTAGTTTATG R: AACTCGCAAACCAAGAGCATAAC	TG	193–205	58	-	5	0.333	0.774	0.728
CL9244.Ct2	F: CTGAGATTTGTTGGTGGGTTTG R: CCAGTATCTCCGAACCACCTCT	AGG	373–382	56	Glutaredoxin 4 isoform 1, *Theobroma cacao*	3	0.438	0.551	0.457
CL8609.Ct2	F: GGAGCTGAATTAGAGCATTAGAGG R: GAAATCTCTCTTGTTCAATCCACC	GA	202–212	55	-	6	0.458	0.663	0.626
Ug13288	F: AGCATTACATTATCCCTTCCTCAC R: CAGAGACGGTGTCGTATTGGA	TAA	240–258	55	peptide chain release factor 1-like, *Glycine max*	5	0.533	0.710	0.649
Ug19883	F: GAGTTATGAATGACGCTACACGAG R: GCCTGCTTTGCGTTTCTTC	TGC	351–360	55	-	3	0.319	0.274	0.240
Ug13288	F: CATCGCCGCCTTTCATAGAC R: GACGCTTAGAATTGGAAGATGATG	TAA	242–263	55	peptide chain release factor 1-like, *Glycine max*	7	0.575	0.758	0.713
Ug31697	F: CAACAGAAAGCACCAACCCAG R: GCATCCACCCTGTTCAGCAT	CTC	241–253	60	-	5	0.729	0.770	0.722
Ug4409	F: CATCGGCCTCTGCTCATACAC R: CGCTTCAGGCTCTCATATTCAG	TCA	272–275	55	-	2	0.292	0.399	0.317
CL12118.Ct3	F: CAGAGAGAATAATAGCAGCCATAG R: CCCAAGCATCCAACAATAAC	AG	289–299	55	Ethylene-responsive transcription factor, *Morus notabilis*	5	0.313	0.349	0.331
Ug31697	F: GGAGGTGATGGAGAAGGTGAGA R: CAACCCATCACAATCTCACATCA	GA	302–312	59	-	4	0.458	0.527	0.459
Mean						4.471	0.418	0.587	0.531

Ug, Cl, Ct, PS, RM, AS, T_a_ and PF represent unigene, cluster, contig, primer sequence, repeat motif, allele size, annealing temperature and putative function respectively; “-” represents no blast hits with known proteins deposited in the public databases.

## Supplementary Materials

The following are available online at https://www.mdpi.com/2073-4425/11/3/322/s1: Table S1: Summary of the identified polymorphic SSRs by CandiSSR. Table S2: Summary of the location information of the identified polymorphic SSRs. Table S3: GO functional annotation of the polymorphic SSR containing sequences. Table S4: KEGG functional classification of the SSR containing sequences. Table S5: Primers for the polymorphic SSRs identified by CandiSSR.
